# Antimicrobial Resistance and Molecular Epidemiology of *Escherichia coli* From Bloodstream Infection in Shanghai, China, 2016–2019

**DOI:** 10.3389/fmed.2021.803837

**Published:** 2022-01-10

**Authors:** Shuzhen Xiao, Chenyue Tang, Qian Zeng, Yilun Xue, Qing Chen, Erzhen Chen, Lizhong Han

**Affiliations:** ^1^Department of Laboratory Medicine, Ruijin Hospital, Shanghai Jiao Tong University School of Medicine, Shanghai, China; ^2^Department of Clinical Microbiology, Ruijin Hospital, Shanghai Jiao Tong University School of Medicine, Shanghai, China; ^3^Faculty of Medical Laboratory Science, Ruijin Hospital, School of Medicine, Shanghai Jiao Tong University, Shanghai, China; ^4^Department of Emergency, Ruijin Hospital, Shanghai Jiao Tong University School of Medicine, Shanghai, China

**Keywords:** bloodstream infection, multilocus sequence typing, molecular epidemiology, resistance gene, *escherichia coli*

## Abstract

**Background:** Bloodstream infections are recognized as important nosocomial infections. *Escherichia coli* (*E. coli*) is the most prevalent Gram-negative bacillary pathogen causing bloodstream infections (BSIs). This retrospective study investigated drug susceptibility and molecular epidemiology of *E. coli* isolated from patients with BSI in Shanghai, China.

**Methods:** We collected *E. coli* isolated from the blood cultures of patients with BSI between January 2016 and December 2019. We randomly selected 20 strains each year to investigate antimicrobial resistance, resistance genes, and molecular epidemiological characteristics. Antimicrobial susceptibility testing was performed by the disk diffusion method. PCR was performed to detect extended-spectrum β-lactamases (ESBLs), carbapenemase genes, and housekeeping genes, and phyloviz was applied to analyze multilocus sequence typing (MLST).

**Results:** Penicillins, first- and second-generation cephalosporins and fluoroquinolones have high resistance rates (>60%). Among the 80 randomly selected strains, 47 (58.8%) produced ESBLs, and one produced carbapenemase. Sequencing of resistance genes identified *bla*_CTX−M−14_ (34%, 16/47), *bla*_CTX−M−15_ (23.4%, 11/47) and *bla*_CTX−M−27_ (14.8%, 7/47) as the most prevalent genotypes of ESBLs. ST131 (14/80) was the most prevalent sequence type (ST), followed by ST1193 (10/80), ST648 (7/80).

**Conclusions:** Our findings suggest that amikacin, carbapenems, and piperacillin-tazobactam have relatively low resistance rates and may be the preferred antibiotic regimens for empiric therapy. ST131 and *bla*_CTX−M−14_ are still the main prevalent in Shanghai with a rapid increase in the occurrence of ST1193 is rapidly increasing and more diverse *bla*_CTX_ genes.

## Introduction

Bacterial bloodstream infections are of global concern (1, 2). They have been proven to be associated with high mortality rates, and are always accompanied by a prolonged hospital stay (3–5). A prospective, multicenter cohort study in 162 intensive care units (ICUs) from 24 countries shows a higher mortality rate in patients with bacterial bloodstream infections (BSI) at the time of admission and who did not receive appropriate treatment (6).

*Escherichia coli* was the most frequent Gram-negative bacterial pathogen according to a study of 264,901 BSIs collected from more than 200 medical centers in 45 countries between 1997 and 2016 (7). The incidence of *E. coli* bloodstream infections (EC-BSI) was reported to have increased by 76% from 2011 to 2015 in the United Kingdom (8). Similar to other countries, *E. coli* also was the most frequent isolate causing BSI in China, accounting for 21.93% of all isolates (9). On the other hand, antimicrobial resistance is more serious in China than in other countries, which makes clinical treatment more challenging (7–10). The proportion of extended-spectrum β-lactamase (ESBL) producing *E. coli* increased to 56.1% in 2018 (11). The key point in the treatment of EC-BSI is the timely and accurate use of antibiotics (12). While conventional bacterial culture and antimicrobial sensitivity test results are time-consuming, treatment of EC-BSI is more dependent on empirical regimens (13). In this study, we aimed to investigate antimicrobial resistance, resistance genes, and their relationship with the phylogenetic group and sequence type (ST) of causative bacterial pathogens isolated from BSI in Shanghai in the current clinical practice.

## Materials and Methods

### Study Setting

This retrospective study was derived from Ruijin Hospital, affiliated with Shanghai Jiaotong University School of Medicine, in Shanghai. Shanghai is the most dynamic city in China, joining the great minds to explore and discover more for human health. In the past 114 years, Ruijin Hospital has been relentlessly innovating in clinical services and medical research as the #1 nation-recognized public teaching hospital in Eastern China. The hospital is a large-scale institution integrated with emergency, outpatient, and inpatient departments (including Obstetrics and Gynecology, Pediatrics, and other basic departments), and medico-technical departments. It serves a population of more than 4,000,000 every year. In addition, the Department of Clinical Microbiology routinely retains Gram-negative bacilli isolated from blood cultures.

This study was approved by the Ethics Committee at Ruijin Hospital affiliated with the School of Medicine at Shanghai Jiao Tong University. This was a retrospective study, performing molecular profiling on bacteria and thus, did not have any study procedure affecting the patient's safety and well-being adversely. A review by the Ethics Committee waived the need to request informed consent from patients. The Ethics Committee number is KY2019-147.

### Bacterial Isolates

A total of 494 non-duplicate *E. coli* isolates (122 in 2016, 89 in 2017, 115 in 2018, and 121 in 2019) were obtained from 494 patients from January 2016 to December 2019. Moreover, 20 isolates were drawn from all samples each year using the random number generation function in Microsoft Office Excel 2010 (Microsoft Corporation, Redmond, WA, USA). Isolates were routinely grown on Columbia blood agar plates and incubated overnight at 35°C. All isolates were identified by matrix-assisted laser desorption ionization-time of flight mass spectrometer (bioMérieux, Marcyl'Étoile, France) and stored in broth containing 30% glycerol at −80°C before they were used.

### Antimicrobial Susceptibility Testing and Screening and Confirmatory Test for ESBLs

Antimicrobial susceptibility was examined by the disk diffusion method. The experiment trial involved 18 antibiotics including the following: cefazolin (30 μg), cefuroxime (30 μg), ceftazidime (30 μg), cefotaxime (30 μg), ceftriaxone (5 μg), cefepime (30 μg), ampicillin (10 μg), aztreonam (30 μg), meropenem (10 μg), imipenem (10 μg), amikacin (30 μg), gentamicin (10 μg), ciprofloxacin (5 μg), tobramycin (10 μg), levofloxacin (5 μg), trimethoprim-sulfamethoxazole (1.25/23.75 μg), piperacillin-tazobactam (100/10 μg), and ampicillin-sulbactam (10/10 μg). All these disks were purchased from Thermo Fisher Scientific (Waltham, MA, USA). *E. coli* ATCC 25922, *Pseudomonas aeruginosa* ATCC 27853, and *Klebsiella pneumoniae* ATCC 700603 were used as quality control strains in the antibiotics susceptibility assay, and the results were interpreted according to CLSI 2020 (14). Screening test for ESBL production was done with ceftazidime and cefotaxime, while imipenem and meropenem were used to screen for carbapenem-resistant strains according to CLSI 2020 (14). The double-disk synergy test (ceftazidime, cefotaxime, ceftazidime-clavulanate, and cefotaxime-clavulanate) was used as a confirmatory test for ESBL producers (14).

### Detection of Resistance Genes

For ESBLs-producing strains, we amplified the following associated resistance genes: *bla*_TEM_, *bla*_SHV_, *bla*_CTX−M−1group_, *bla*_CTX−M−9group_, *bla*_GES_, *bla*_PER_, *bla*_VEB_, and *bla*_OXA(−1,−2,−10group)_; for carbapenem-resistant strains, the following resistance genes were amplified: *bla*_OXA−48group_, *bla*_VIM_, *bla*_IPM_, *bla*_KPC_, and *bla*_NDM_. Primer was shown in Table 1. All positive products were sequenced using ABI3730xl DNA Analyzer by MAP Biotech Shanghai, China. The types of ESBLs and carbapenemase genes were determined by comparing the sequences in GenBank (http://www.ncbi.nlm.nih.gov/BLAST) National Center for Biotechnology Information (U.S. National Library of Medicine, Bethesda, MD, USA).

**Table 1:** Sequences of primers for resistance genes PCR amplification.

**Genes**	Primes^*^	**Prime sequences (5'−3')**	**Expected amplicon size (bp)**
TEM	TEM-F	ATAAAATTCTTGAAGACGAAA	1,080
	TEM-R	GACAGTTACCAATGCTTAATC	
SHV	SHV-F	TGGTTATGCGTTATATTCGCC	865
	SHV-R	GGTTAGCGTTGCCAGTGCT	
CTX-M-1	CTX1-F	AAAAATCACTGCGCCAGTTC	415
	CTX1-R	AGCTTATTCATCGCCACGTT	
CTX-M-9	CTX9-F	CAAAGAGAGTGCAACGGATG	205
	CTX9-R	ATTGGAAAGCGTTCATCACC	
GES	GES-F	ATGCGCTTCATTCACGCAC	846
	GES-R	CTATTTGTCCGTGCTCAGG	
PER	PER-F	AGTCAGCGGCTTAGATA	978
	PER-R	CGTATGAAAAGGACAATC	
VEB	VEB-F	GCGGTAATTTAACCAGA	961
	VEB-R	CGTATGAAAAGGACAATC	
OXA-1	OXA1-F	CTGTTGTTTGGGTTTCGCAAG	440
	OXA1-R	CTTGGCTTTTATGCTTGATG	
OXA-2	OXA2-F	CAGGCGCYGTTCGYGATGAGTT	233
	OXA2-R	GCCYTCTATCCAGTAATCGCC	
OXA-10	OXA10-F	GTCTTTCRAGTACGGCATTA	822
	OXA10-R	GATTTTCTTAGCGGCAACTTA	
OXA-48	OXA48-F	ATGCGTGTATTAGCCTTATC	781
	OXA48-R	CTAGGGAATAATTTTTTCCT	
VIM	VIM-F	TCTACATGACCGCGTCTGTC	953
	VIM-R	TGTGCTTTGACAACGTTCGC	
IMP	IMP-F	AACCAGTTTTGCCTTACCAT	520
	IMP-R	CTACCGCAGCAGAGTCTTTG	
KPC	KPC-F	TTACTGCCCGTTGACGCCCAATCC	720
	KPC-R	TCGCTAAACTCGAACAGG	
NDM	NDM-F	GCCATGTCACTGAATACTCGT	815
	NDM-R	GCGATCCTTCCAACTCGT	

* *Primer, the “F” meant the forward primer and the “R” meant the reverse primer*.

### Analysis of the Genetic Relationship of the Isolates

The experiment further involved seven housekeeping genes of *E. coli* (*adk, fumC, gyrB, icd, mdh, purA*, and *recA*) which were amplified according to the MLST database (https://pubmlst.org). The seven housekeeping genes of each strain were compared online to obtain the ST (15). MLST analysis was performed using phyloviz. Strains were grouped by the phyloviz algorithm. A rough sketch was drawn to show the genetic relationships by using user-specified group definitions based on their allelic characteristics (16).

### Statistical Analysis

We expressed continuous variables as mean ± *SD* or median and interquartile range if not normal distributed, and categorical variables were compared using chi-square test and Fisher's exact test whenever applicable. Two-tailed *P*-values < 0.05 were statistically significant. All statistical analysis were performed using SPSS 23.0 (IBM, Armonk, NY, USA).

## Results

### Distribution Characteristics of Patients and Strains

The age of the 80 patients ranged from 17 to 87 years, with quartiles ranging from 53 to 74 years. There were more men (57.5%, 46/80) than women (42.5%, 34/80). These patients were mainly from the pancreatic surgery (13/80), gastrointestinal surgery (8/80), intensive care unit (5/80), and infection department (5/80).

### Antimicrobial Resistance

The results showed that 47 isolates (58.8%, 47/80) were ESBLs-producing, and three isolates (3.75%) were resistant to carbapenems. Among all isolates, the highest resistance rates were to ampicillin (85%), cefazolin (72.5%), cefuroxime (68.5%), cefotaxime (63.7%), ceftriaxone (62.5%), ciprofloxacin (61.3%), levofloxacin (60%), and trimethoprim-sulfamethoxazole (57.5%). In contrast, resistance rates to imipenem (1.3%), meropenem (3.8%) and piperacillin-tazobactam (5%) were low. In addition, no amikacin-resistant strains were found. There was a significant difference in resistance to cephalosporins between ESBL-producing and non-ESBL-producing isolates (*P* < 0.05) (Table 2).

**Table 2:** Antimicrobial resistance of Eighty *E. coli* isolates from bloodstream infections.

**Antimicrobial agents**	Number of isolates (%)								
	**2016**	**2017**	**2018**	**2019**	**P1**	ESBL (*n* = 47)^*^	**non-ESBL (*n* = 33)**	**P2**	**Total (*n* = 80)**
Cefazolin	12 (60.0)	15 (75.0)	17 (85.0)	14 (70.0)	0.263	47 (100)	11 (33.3)	<0.001	58 (72.5)
Cefuroxime	12 (60.0)	15 (75.0)	14 (70.0)	14 (70.0)	0.741	46 (97.9)	9 (27.8)	<0.001	55 (68.8)
Ceftazidime	1 (5.0)	4 (20.0)	2 (10.0)	1 (5.0)	0.503	7 (14.9)	1 (3.0)	0.082	8 (10.0)
Cefotaxime	10 (50.0)	14 (70.0)	14 (70.0)	13 (65.0)	0.707	46 (97.9)	5 (15.2)	<0.001	51 (63.8)
Ceftriaxone	10 (50.0)	14 (70.0)	13 (65.0)	13 (65.0)	0.190	45 (95.7)	5 (15.2)	<0.001	50 (62.5)
Cefepime	2 (10.0)	4 (20.0)	3 (15.0)	4 (20.0)	0.157	12 (25.3)	1 (3.0)	0.007	13 (16.3)
Ampicillin	15 (75.0)	18 (90.0)	18 (90.0)	17 (85.0)	0.629	47 (100)	21 (63.6)	<0.001	68 (85.0)
Aztreonam	4 (20.0)	5 (20.0)	7 (35.0)	3 (15.0)	0.741	17 (36.2)	2 (6.1)	0.002	19 (23.8)
Meropenem	0 (0)	1 (5.0)	1 (5.0)	1 (5.0)	0.792	2 (4.3)	1 (3.0)	0.776	3 (3.8)
Imipenem	0 (0)	0 (0)	1 (5.0)	0 (0)	0.417	1 (2.1)	0 (0)	0.23	1 (1.3)
Amikacin	0 (0)	0 (0)	0 (0)	0 (0)	/	0 (0)	0 (0)	/	0 (0)
Gentamicin	9 (45.0)	7 (35.0)	8 (40.0)	7 (35.0)	0.719	20 (42.6)	11 (33.3)	0.405	31 (38.8)
Ciprofloxacin	9 (45.0)	15 (75.0)	13 (65.0)	12 (60.0)	0.256	31 (66.0)	18 (54.6)	0.302	49 (61.3)
Tobramycin	4 (20.0)	5 (25.0)	6 (30.0)	4 (20.0)	0.782	14 (29.8)	5 (15.2)	0.13	19 (23.8)
Levofloxacin	8 (40.0)	15 (75.0)	13 (65.0)	12 (60.0)	0.144	31 (66.0)	17 (51.5)	0.194	48 (60.0)
Trimethoprim-sulfamethoxazole	11 (55.0)	11 (55.0)	11 (55.0)	13 (65.0)	0.731	31 (66.0)	15 (45.5)	0.068	46 (57.5)
Piperacillin-tazobactam	0 (0)	1 (5.0)	1 (5.0)	2 (10.0)	0.525	3 (6.4)	1 (3.0)	0.498	4 (5.0)
Ampicillin-sulbactam	4 (20.0)	7 (35.0)	2 (10.0)	2 (10.0)	0.363	9 (19.2)	6 (18.2)	0.913	15 (18.8)

* *ESBL (n = 47), if the ESBL confirmatory test was positive, we defined the isolate as an ESBL-producer and the negative as a non-producer*.

### Resistance Genes

In the confirmatory test for ESBLs, 47 isolates were positive. Among the 47 isolates of ESBL-producing strains, CTX-M-14 (14, 29.8%), CTX-M-15 (8, 17.0%), and CTX-M-27 (7, 14.9%) were the most frequent ESBLs (Table 3). While CTX-M-55 had a relatively low prevalence of 12.8% (ranked the fourth). Most of the ESBLs-producing strains (41/47) only carried one ESBL gene, while the others (6/47) carried two or more ESBL genes. Especially in ST131, four strains (4/12) carry two or more ESBL genes. The prevalence rates of β-lactamase type TEM-1 and OXA-1 were 44.7% (21/47) and 12.8% (6/47). No *bla*_CTX−M(−2,−8,−25group)_, *bla*_VEB_, *bla*_GES_, *bla*_OXA−2group_, or *bla*_PER_ genes were found. Among the three carbapenem-resistant strains, one harbored the *bla*_NDM_ gene, and the other two strains harbored the *bla*_CTX−M−15_ gene.

**Table 3:** Resistant genes in ESBL-producing isolates from *E. coli* bloodstream isolates from 2016 to 2019.

**Genes**	Number of isolates (%)				
	**2016**	**2017**	**2018**	**2019**	**Total**
**ESBL**	8 (40.0)	13 (65.0)	13 (65.0)	13 (65.0)	47 (58.8)
*bla*_CTX−M−3_	1 (12.5)	0 (0.0)	0 (0.0)	0 (0.0)	1 (2.1)
*bla*_CTX−M−14_	1 (12.5)	5 (38.5)	6 (46.2)	4 (30.8)	16 (34.0)
*bla*_CTX−M−15_	3 (37.5)	3 (23.1)	2 (15.4)	3 (23.1)	11 (23.4)
*bla*_CTX−M−27_	0 (0.0)	2 (15.4)	0 (4.0)	5 (38.5)	7 (14.9)
*bla*_CTX−M−55_	0 (0.0)	2 (15.4)	4 (30.8)	0 (0.0)	6 (12.8)
*bla*_CTX−M−199_	0 (0.0)	0 (0.0)	0 (0.0)	1 (7.7)	1 (2.1)

### Multilocus Sequence Typing

**Figure 1:**
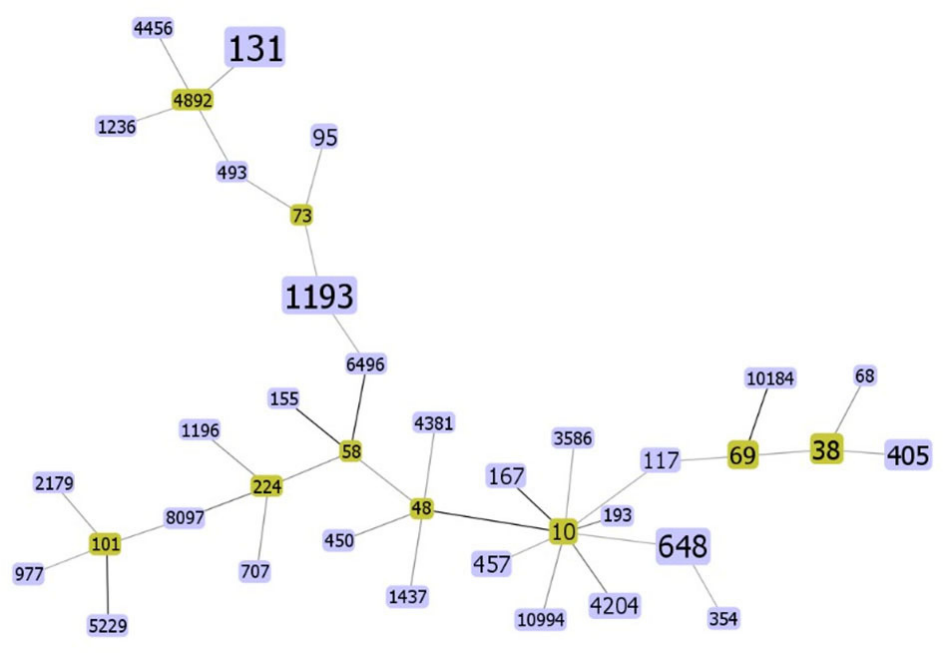
The rough sketch produced by phyloviz with the SLV (default) group definition, representing 80 *E. coli* isolates from the bloodstream: there are 38 STs. The yellow rectangle indicates putative founder and the area of each rectangle means the prevalence of the ST in the MLST data of this study. The darker the color of the connecting lines, the more closely related they are.

Multilocus sequence typing (MLST) analysis distinguished 38 different STs into 9 non-overlapping clonal complexes (Figure 1). The distribution of ESBLs among ST is shown in Supplement 1. The most dominant ST was ST131 (14/80), where the percentage of ESBLs-producing strains was 85.7%, with the most kinds of CTX-M-15 (4/11). ST1193 was ranked second (10/80), where the percentage of ESBLs-producers was 80%, which harbored the most CTX-M-27 of all CTX-M-27 (3/7). To our surprise, ST1193 has been 100% resistant to quinolones. In addition, ST69, ST131, and ST405 had the phenomenon of harboring two or more ESBL genes.

## Discussion

Since the beginning of the 21st century, several international studies indicate a significant increase in ESBL-producing EC-BSI (17, 18). For bloodstream infections, third-generation cephalosporins are prescribed most frequently in current practice (19). However, the rate of resistance to third-generation cephalosporins by *E. coli* is also increasing (2). In our study, the resistance rates of ceftazidime, cefotaxime, and ceftriaxone were 10%, 63.7%, and 62.5%, respectively. Cefotaxime and ceftriaxone had such high resistance rates, and therefore, they are not recommended. On the other hand, ceftazidime had a lower resistance rate of 10% and can be considered when combination therapy. Considering the low resistance rate of amikacin, piperacillin-tazobactam, and carbapenems, we recommend switching to these antibiotics as options for the treatment of EC-BSIs.

In this study, the percentage of ESBL was 58.8%, slightly lower than previous studies in Shanghai (20), but still higher than in Denmark (9.1%), Argentina (17%), and Thailand (30%) (21–23). In contrast to previous studies in Shanghai and Tianjin, in which CTX-M-15 was the predominant ESBL (20, 24), we found that CTX-M-14 was the predominant ESBL in this study, suggesting that epidemiological surveys need to be available in all locations and to be monitored continuously. In addition, CTX-M-27 is growing slowly in certain parts of the world (25). The *bla*_CTX−M−27_ is associated with the IncF plasmid. Horizontal transfer is important in the propagation of the *bla*_CTX−M−27_, which was transferred by conjugation (26). IncF plasmids encode many addiction systems that ensure and help maintain antimicrobial resistance determinants and virulence factors even in the absence of antibiotic selection pressure (27). In our study, CTX-M-27 became the third prevalent (14.9%, 7/47), and the rising trend of antibiotic resistance needs to be alerted. Our research identified six strains carrying both CTX-M enzymes and OXA enzymes, especially in strains belonging to ST131. According to the literature, *bla*_CTX_ and *bla*_OXA_ genes are usually carried by plasmids (28), while the clonal spread of ST131 has been reported to promote the prevalence of CTX (29). More research will be carried out to explore the genetic environment of the two genes and the correlation between ST131 and their growing transmission. The carbapenemase resistance gene *bla*_NDM−5_ was detected in one of the three carbapenem-resistant strains, and the other two strains were ESBLs-producing. Two carbapenemases, KPC and NDM, were responsible for phenotypic resistance in 90% of the carbapenem-resistant Enterobacteriaceae (30). Studies unveiled many distinguishing features for their successful persistence and spread (31, 32). And more intense monitoring of those resistant genes is urgently to prevent untreatable infections. It has been demonstrated that reduction of outer membrane permeability, the combination of porin loss with ESBLs, and the overexpression of efflux pumps can contribute to carbapenem resistance (33). The resistance mechanisms of the two strains in this study need more investigation.

Multilocus sequence typing (MLST) is a common tool used for genotype-specific bacteria (34). The method has long been widely available internationally to monitor cloning and evolutionary studies and to propose common ancestral lineages among bacteria. Our study showed the highest proportion of ST131 (14/80), which is also consistent with previous findings (35). Unlike earlier studies (36), ST1193 rather than ST405 was the second most prevalent sequence type in this study. ST1193 *E. coli* has increased dramatically in recent years and is emerging as a new, virulent, and resistant spectrum of fluoroquinolone-resistant *E. coli* (37, 38), and our antimicrobial sensitivity tests suggested that ST1193 had a much higher rate of quinolone resistance than the other sequence type *E. coli*. Furthermore, ST1193 also had a high resistant rate to cephalosporins and ampicillin and the percentage of ESBLs-producers was 80.0%. It has been reported that ST1193 was similar to ST131 in pathogenicity- and survivability-associated phenotypic characteristics (37) and its increase may be related to horizontal transfer of epidemic plasmid IncI1/ST16 (39). We need to pay attention to the wide spread of ST1193 and prevent the mass clonal spread of ST1193 like ST131. The diversity of STs also illustrated the absence of clonal transmission of EC-BSI in Shanghai.

There were limitations to this study. For one, the surveillance data based on just one hospital may not be generalizable enough or extrapolated directly to the whole region. In China, most hospitals do not save clinical isolates routinely. Further studies are needed to evaluate more hospitals to determine more actual characteristics of *E. coli* from bloodstream infections in Shanghai, even in China. Also, the study analyzed *E. coli* based on traditional methods, including the disk diffusion method for antimicrobial susceptibility tests and PCR for resistance genes.

In conclusion, for empirical therapy in EC-BSI, amikacin, piperacillin-tazobactam, and carbapenems may be the preferred antibiotic regimens. While penicillins, first- and second-generation cephalosporins, and fluoroquinolones should be avoided in treating EC-BSI. Even CTX-M-14 is still the main prevalent, the distribution of CTX enzymes is more diverse and CTX-M-27 has a significant increase. We need to be alert to the rising isolation rate of ST1193 and the high rate of antibiotic resistance.

## Data Availability Statement

The original contributions presented in the study are included in the article/supplementary material, further inquiries can be directed to the corresponding author/s.

## Author Contributions

LH, EC, QZ, and QC: conceptualization. SX and CT: data curation. SX, CT, and YX: formal analysis. LH and EC: funding acquisition. LH: resources. CT: writing—original draft preparation. SX: writing—review and editing. All authors contributed to the article and approved the submitted version.

## Funding

We acknowledge the support by Program of Shanghai Jiao Tong University School of Medicine (DLY201803), Major Clinical Research Project of Shanghai Hospital Development Center (SHDC2020CR1028B), Scientific and Technological Innovation Act Program of Science and Technology Commission of Shanghai Municipality (18411950900), and Nature Science Foundation of China (81772107).

## Conflict of Interest

The authors declare that the research was conducted in the absence of any commercial or financial relationships that could be construed as a potential conflict of interest.

## Publisher's Note

All claims expressed in this article are solely those of the authors and do not necessarily represent those of their affiliated organizations, or those of the publisher, the editors and the reviewers. Any product that may be evaluated in this article, or claim that may be made by its manufacturer, is not guaranteed or endorsed by the publisher.
